# HIFU immunotherapy: lessons from animal to clinical studies

**DOI:** 10.1186/2050-5736-3-S1-O40

**Published:** 2015-06-30

**Authors:** Feng Wu

**Affiliations:** 1 University of Oxford, Oxford, United Kingdom

## Background/introduction

The ideal cancer therapy not only induces the death of all localized tumor cells, but also activates a systemic antitumor immunity. High intensity focused ultrasound (HIFU) has the potential to be such a treatment, as it can non-invasively ablate a targeted tumor below the skin surface, and may subsequently augment host antitumor immunity.

## Methods

This talk is to introduce increasing animal and clinical evidences linking antitumor immune response to HIFU ablation, review the potential mechanisms, and discuss challenges and opportunities involved in HIFU-enhanced host antitumor immunity.

## Results and conclusions

It is concluded that HIFU immunotherapy may play an important role in preventing local recurrence and metastasis of cancer after HIFU treatment.

